# Video Game Addiction and its Relationship with Sleep Quality among Medical Students

**DOI:** 10.1007/s44197-024-00265-x

**Published:** 2024-06-19

**Authors:** Faisal Ahmed Dakheel-allah Alghamdi, Faisal Ahmed Ghanim Alghamdi, Abdullah Abusulaiman, Abdulaziz Jabr Alsulami, Mohammed Bamotref, Albraa Alosaimi, Omar Bamousa, Siraj Omar Wali

**Affiliations:** 1 Faculty of Medicine, King Abdulaziz University, Jeddah, Saudi Arabia; 2 College of Medicine, Sleep Medicine and Research Center, Consultant in Pulmonary & Sleep Medicine, King Abdulaziz University, King Abdulaziz University Hospital, PO BOX 21589, Jeddah, 80215 Saudi Arabia

**Keywords:** Students, Medical, Gaming, Addiction, Abnormal, Sleep Quality

## Abstract

**Background:**

Although many health problems, including sleep disorders, have been associated with video gaming, further studies are required to establish the validity of these connections. This study aimed to determine the prevalence of gaming addiction among medical students and its association with poor sleep quality, which may be reflected in academic performance.

**Method:**

A cross-sectional survey was conducted between January and June 2023 among medical students at the institution under study. An online survey was conducted and was divided into three sections. The first section included the demographic data, the second section included the 7-item Gaming Addiction Scale (GAS), and the third section included the Pittsburgh Sleep Quality Index. Using the GAS, and based on the total score, gamers were classified as addicted, problematic, engaged, or normal. Hence, abnormal gamers include engaged, problematic, and addicted gamers.

**Result:**

There were 356 participants with a mean age of 22.5 -/+ 1.8 years, and 75.3% were males. The data showed that 38.8% of the study population were abnormal gamers: 40 (11.2%) engaged gamers, 81 (22.8%) problematic gamers, and 17 (4.8%) addicted gamers. Furthermore, abnormal gaming was linked to poor sleep quality when comparing abnormal gamers with normal gamers (92% vs. 80.3%, *p* = 0.002). Further comparison between the types of abnormal gamers revealed that addicted gamers were found to rely on sleep medication to help them sleep at night and took longer time to fall asleep (*p* = 0.050 and *p* = 0.045, respectively).

**Conclusion:**

Abnormal gamers are common among medical students and strongly associated with poor sleep quality compared to normal gamers.

## Introduction

Problematic video game playing has grown significantly over the past decade, along with internet addiction, and has caused many public health problems. The Diagnostic and Statistical Manual of Mental Disorders, Fifth Edition (DSM-5), has classified Internet gaming disorder (IGD) as a condition that causes “significant impairment or distress” in several aspects of a person’s life but nevertheless has not recognized it as addictive mental disorder [1]. These findings highlight the need for further research to address this issue.

Neurobiological studies have demonstrated that video games affect the brain. More specifically, when players receive rewards in a game, they trigger the release of dopamine in the reward center of the brain, which is linked to feelings of pleasure [2, 3]. This may explain why some gamers spend more time playing games and lose interest in real-life activities. Problematic video game playing is also associated with stress and poor academic performance. A recent study conducted in Saudi Arabia, Al-Qassim region, found that women addicted to video games had high stress levels, poor school grades, unhealthy dietary habits, and inactive lifestyles [4]. Problematic video game playing influences sleep quality by disturbing the sleep-wake cycle, leading to insomnia [5]. Multiple studies have shown that individuals with problematic video game playing experience poor sleep quality, excessive daytime sleepiness, and poor sleep habits [6–8]. Furthermore, disturbances in the sleep-wake cycle have been reported in players who play online video games that allow players in different time zones to join and play simultaneously, meaning that some players stay awake late at night to play with others [6, 9].

Although the negative effects of problematic video gaming on sleep quality have been widely studied, such studies are lacking in Saudi Arabia. Considering the significant increase in video game sales in this region and worldwide, it is important to determine the prevalence of gaming addiction and its association with poor sleep quality among young people. While there have been many previous studies in other countries using the old classification of gaming addiction (addicted or not addicted), we believe that the new classification provides a better perspective and clearly distinguishes between the different types of gaming addiction. This provides additional parameters for comparison, which aids in determining the type of gaming addiction associated with poor sleep quality.

This study aimed to address video game addiction and its impact on sleep patterns among young adults in a medical student community in Saudi Arabia.

## Methods

A cross-sectional online survey that was conducted on medical students at the college of medicine, King Abdulaziz University. The survey was distributed among medical students of all levels using social media platforms (WhatsApp and Telegram) over 6 months period between January and June 2023. Each member of the research team was assigned to the students of a certain academic year to send the survey and follow up their reply to ensure adequate response rate by encouraging them to participate and solving all inquiries regarding the survey. There were 356 participants with complete data giving a response rate of 29.75% (356 of 1200).

Sample Size: According to our student’s affair we have 1200 students in the medical faculty in 2022/2023, so when calculating the sample size, with a CI of 95% and a 5% margin of error we get *n* = 292.

The survey is divided into three sections. The first section collected demographic data (age, sex, academic year, and social status). The second section included the 7-item gaming addiction scale (GAS) [10], and the third section included the Pittsburgh Sleep Quality Index (PSQI) [11].

### Instruments

#### Gaming Addiction Scale **GAS** [10]

GAS was developed by Lemmens et al., and consists of seven questions that measure different aspects of gaming addiction over a 6-months period. The items include salience/preoccupation (thinking all day long about playing games), tolerance (playing longer than intended), mood modification (playing games to forget about real life), relapse (unsuccessfully trying to reduce the time spent on games by oneself or others), withdrawal (feeling upset when unable to play), conflict (arguments with others), and problems (neglecting important activities to play games). Each question is rated on a scale of 1 (never) to 5 (very often). Based on the total score, gamers are classified as addicted (scoring 3 or more on the four final items), problematic (scoring 3 or more on two or three of the four final items), engaged (scoring three or more on the first three items but not scoring 3 or above on more than one of the final four items), or normal (not classified as addicted, problematic, or engaged gamers) [12, 13].

#### Pittsburgh Sleep Quality Index **PSQI** [11]

The PSQI was used to evaluate the sleep quality. This tool consists of 19 questions addressing various factors related to sleep quality in the past month such as subjective sleep quality, sleep latency, sleep duration, habitual sleep efficiency, sleep disturbances, use of sleeping medication, and daytime dysfunction. Each question is rated on a scale of 0–3, with higher scores indicating more significant sleep problems. The total score is obtained by adding the scores of the seven sections and ranged from 0 to 21. Higher scores indicate greater sleep difficulties. Individual sleep quality can be described using PSQI scores. A score of 5 or higher indicates poor sleep quality, while a score of less than 5 indicates good sleep quality.

### Statistical Analysis

The data are expressed as frequency (%) for categorical data and as mean ± standard deviation (minimum-maximum) and median (25th–75th percentile) for parametric data. Data were analyzed using SPSS version 22 (Statistical Package for Social Sciences, IBM Corp., Armonk, New York). The Shapiro–Wilk test was used to determine whether the value distributions were normal. Pearson’s chi-square test of association was used to examine the associations between the study variables and the significance between the categorized variables. Mann–Whitney tests were performed to explore parametric differences between the two groups, and the Kruskal–Wallis test was used for comparisons between more than two groups. Binary logistic regression analysis was used to assess risk factors for gaming addiction and poor sleep quality. A *p*-value *<* 0.05 was considered statistically significant.

## Results

A total of 356 students predominantly males, (75.3%) were recruited for the study. The participants’ ages ranged from 18 to 29 years, with a mean age of 22.5 years. Majority of the participants (76.2%) were in clinical years (i.e., 4th, 5th, and 6th years) (Table 1).

**Table 1:** Demographic characteristics of participants (*n* = 356)

Characteristics	Value
**Age** (years)	22.50 ± 1.78 (18.00–29.00)
**Sex**	
Men	268 (75.3%)
Women	88 (24.7%)
**Academic year**	
Second Year	48 (13.5%)
Third Year	37 (10.4%)
Fourth Year	65 (18.3%)
Fifth Year	95 (26.7%)
Sixth Year	111 (31.2%)
**Type of housing**	
Villa	185 (52.0%)
Apartment	155 (43.5%)
Traditional house	14 (3.9%)
Other types of housing	2 (0.6%)
**Tenure of housing**	
Owned	277 (77.8%)
Rented	77 (21.6%)
Provided	2 (0.6%)
**Chronic diseases**	
No	326 (91.6%)
Yes	30 (8.4%)

A total of 218 **normal gamers** (61.2%), 40 **engaged gamers** (11.2%), 81 **problem gamers** (22.8%), and 17 (4.8%) **addicted gamers** were observed (Table 2).

**Table 2:** Game addiction scale (GAS) scores among participants

Addiction criteria	How often in the last 6 months	Endorsed (rated ≥ 3)
**Salience/ preoccupation**	1. Have you thought all day long about playing a game?	168 (47.2%)
**Tolerance**	2. Have you played longer than you intended?	190 (53.4%)
**Mood modification**	3. Have you played games to forget about real life?	141 (39.6%)
**Relapse**	4. Have others unsuccessfully tried to reduce your time spent on games?	74 (20.8%)
**Withdrawal**	5. Have you felt upset when you were unable to play?	101 (28.4%)
**Conflict**	6. Have you had arguments with others (e.g., family, friends) over your time spent on games?	62 (17.4%)
**Problems**	7. Have you neglected important activities (e.g., school, work, and sports) to play games?	97 (27.2%)
**Game addiction category**		
**Normal gamers**	218 (61.2%)	
**Engaged gamers**	40 (11.2%)	
**Problem gamers**	81 (22.8%)	
**Addicted gamers**	17 (4.8%)	

There was a significant increase in the mean score for sleep disturbance (*p* < 0.0001), use of sleep medications (*p* = 0.007), daytime dysfunction (*p* < 0.0001), and global PSQI score (*p* = 0.0001) in abnormal gamers (Table 3a).

**Table 3a:** Mean PSQI item scores and total scores in normal and abnormal gamers

PSQI items	All participants (*n* = 356)	Normal gamers (*n* = 218)	Abnormal gamers (*n* = 138)	Significance
**Subjective sleep quality**				0.067
Mean+/- SD (min- max)	**1.24 ± 0.79 (0–3)**	**1.19 ± 0.79 (0–3)**	**1.33 ± 0.80 (0–3)**	
Median (25–75 interquartile)	**1 (1–2)**	**1 (1–2)**	**1 (1–2)**	
**Sleep latency**				**0.203**
Mean+/- SD (min- max)	**1.68 ± 0.96 (0–3)**	**1.62 ± 1.00 (0–3)**	**1.76 ± 0.89 (0–3)**	
Median (25–75 interquartile)	**2 (1–2)**	**2 (1–2)**	**2 (1–2)**	
**Sleep duration**				**0.256**
Mean+/- SD (min- max)	**1.63 ± 1.08 (0–3)**	**1.58 ± 1.08 (0–3)**	**1.71 ± 1.09 (0–3)**	
Median (25–75 interquartile)	**2 (1–3)**	**2 (1–3)**	**2 (1–3)**	
**Habitual sleep efficiency**				**0.381**
Mean+/- SD (min- max)	**0.89 ± 0.95 (0–3)**	**0.85 ± 0.92 (0–3)**	**0.96 ± 0.995 (0–3)**	
Median (25–75 interquartile)	**1 (0–1)**	**1 (0–1)**	**1 (0–2)**	
**Sleep disturbance**				**0.0001**
Mean+/- SD (min- max)	**1.21 ± 0.55 (0–3)**	**1.12 ± 0.54 (0–3)**	**1.34 ± 0.55 (0–3)**	
Median (25–75 interquartile)	**1 (1–2)**	**1 (1–1)**	**1 (1–2)**	
**Use of sleep medications**				**0.007**
Mean+/- SD (min- max)	**0.37 ± 0.79 (0–3)**	**0.27 ± 0.65 (0–3)**	**0.54 ± 0.95 (0–3)**	
Median (25–75 interquartile)	**0 (0–0)**	**0 (0–0)**	**0 (0–1)**	
**Daytime dysfunction**				**0.0001**
Mean+/- SD (min- max)	**1.16 ± 0.92(0–3)**	**0.98 ± 0.87(0–3)**	**1.44 ± 0.94(0–3)**	
Median (25–75 interquartile)	**1 (0–2)**	**1 (0–2)**	**2 (1–2)**	
**Global PSQI Score**				**0.0001**
Mean+/- SD (min- max)	**8.18 ± 3.50 (0–20)**	**7.61 ± 3.44 (0–18)**	**9.09 ± 3.42 (1–20)**	
Median (25–75 interquartile)	**8 (6–11)**	**8 (5–10)**	**9 (7–11)**	
**Global PSQI Score category**				**0.002**
**Normal sleep quality (< 5)**	**54 (15.2%)**	**43 (19.7%)**	**11 (8.0%)**	
**Poor sleep quality (≥ 5)**	**302 (84.8%)**	**175 (80.3%)**	**127 (92.0%)**	

Parametric data are presented as mean ± standard deviation (minimum-maximum) and median (25th–75th percentiles), and categorized data are presented as the frequency (%). Abnormal gamers included engaged, problematic, and addicted gamers. Addicted gamers showed a significant increase in the mean score of sleep latency (*p* = 0.045) and the use of sleep medications (*p* = 0.050) compared with engaged and problem gamers (Table 3b).

**Table 3b:** Mean PSQI item scores and total scores for abnormal gamers

PSQI items	Engaged gamers (*n* = 40)	Problematic gamers (*n* = 81)	Addicted gamers (*n* = 17)	Significance
**Subjective sleep quality**				0.938
Mean+/- SD (min- max)	1.30 ± 0.65 (0–3)	1.33 ± 0.82 (0–3)	1.41 ± 1.00 (0–3)	
Median (25–75 interquartile)	1 (1–2)	1 (1–2)	1 (1–2)	
**Sleep latency**				0.045*
Mean+/- SD (min- max)	1.63 ± 0.95 (0–3)	1.74 ± 0.88 (0–3)	2.24 ± 0.66 (1–3)	
Median (25–75 interquartile)	1 (1–2)	2 (1–2)	2 (1–3)	
**Sleep duration**				0.971
Mean+/- SD (min- max)	1.73 ± 1.09 (0–3)	1.69 ± 1.10 (0–3)	1.76 ± 1.09 (0–3)	
Median (25–75 interquartile)	2 (1–3)	2 (1–3)	2 (1–3)	
**Habitual sleep efficiency**				0.245
Mean+/- SD (min- max)	1.05 ± 1.09 (0–3)	0.84 ± 0.92 (0–3)	1.29 ± 1.11 (0–3)	
Median (25–75 interquartile)	1 (6)	1 (0–1)	1 (6)	
**Sleep disturbance**				0.091
Mean+/- SD (min- max)	1.20 ± 0.41 (1–2)	1.37 ± 0.58 (0–3)	1.53 ± 0.62 (1–3)	
Median (25–75 interquartile)	1 (1–2)	1 (1–2)	1 (1–2)	
**Use of sleep medications**				**0.050** ^*^
Mean+/- SD (min- max)	0.40 ± 0.90 (0–3)	0.51 ± 0.90 (0–3)	1.00 ± 1.18 (0–3)	
Median (25–75 interquartile)	0 (0–0)	0 (0–1)	1 (6)	
**Daytime dysfunction**				0.470
Mean+/- SD (min- max)	1.28 ± 0.96 (0–3)	1.51 ± 0.92 (0–3)	1.53 ± 0.94(0–3)	
Median (25–75 interquartile)	1 (6)	2 (1–2)	2 (1–2)	
**Global PSQI score**				0.179
Mean+/- SD (min- max)	8.58 ± 3.50 (1–17)	8.99 ± 3.13 (3–16)	10.76 ± 4.01 (4–20)	
**Poor sleep quality (PSQI ≥ 5) N (%)**	35 (87.5%)	76 (93.8%)	16 (94.1%)	0.45

In the binary logistic regression analysis **(**Table 4**)**, the following factors among gamers with poor sleep quality had a significant independent positive association with abnormal gaming: mood modification (Exp(B): 1.565, 95% confidence interval [CI]: 1.017–2.407, *p* = 0.041); conflict (Exp(B): 2.041, 95% CI: 1.259–3.307, *p* = 0.004); and problem gamers (**Exp**(B): 3.735, 95% CI: 1.424–9.798, *p* = 0.007), while relapse was associated with a lower risk of abnormal gaming (Exp(B): 0.487, 95% CI: 0.286–0.827, *p* = 0.008).

**Table 4:** Binary logistic regression model capturing independent predictors of abnormal gaming among demographic characteristics and GAS items in gamers with poor sleep quality

Items	Exp (B)	95% CI	*P*-value
**Age** (years)	0.951	0.798–1.133	0.571
**Sex**			
Men	0.974	0.481–1.970	0.941
Women	Reference		
**Type of housing**			
Villa	Reference		
Apartment	1.997	0.988–4.036	0.054
Traditional house	0.596	0.156–2.271	0.448
Other types of housing	0.418	0.00–0.00	0.999
**Tenure of housing**			
Owned	Reference		
Rented	0.973	0.401–2.311	0.9151
Provided	0.177	0.010–3.132	0.238
**Chronic diseases**			
No	References		
Yes	1.677	0.480–5.861	0.418
**GAS**			
**Salience/preoccupation**	1.015	0.677–1.524	0.941
**Tolerance**	1.180	0.792–1.757	0.416
**Mood modification**	1.565	1.017–2.407	0.041*
**Relapse**	0.487	0.286–0.827	0.008**
**Withdrawal**	1.311	0.791–2.173	0.294
**Conflict**	1.123	0.698–1.807	0.632
**Problems**	2.041	1.259–3.307	0.004**

In the binary logistic regression analysis (Table 5), the following factors had a significant independent positive association with poor sleep quality: sleep disturbance (Exp(B): 2.112, 95% CI: 1.408–3.169, *p* < 0.0001); use of sleep medications (Exp(B): 1.526, 95% CI: 1.160–2.008, *p* = 0.003); daytime dysfunction (Exp(B): 1.766, 95% CI: 1.381–2.257, *p* < 0.0001) and poor sleep (Exp(B): 2.837, 95% CI: 1.408–5.716, *p* < 0.0001).

**Table 5:** Binary logistic regression model capturing independent predictors of poor sleep quality among demographic characteristics and PSQI items in abnormal gamers

Items	Exp (B)	95% CI	*P*-value
**Age** (years)	0.971	0.740–1.273	0.830
**Sex**			
Men	0.991	0.406–2.417	0.983
Women	Reference		
**Type of housing**			
Villa	Reference		
Apartment	1.985	0.972–4.054	0.060
Traditional house	0.611	0.159–2.349	0.474
Other types of housing	0.418	0.00–0.00	0.999
**Tenure of housing**			
Owned	Reference		
Rented	0.955	0.401–2.278	0.918
Provided	0.171	0.010–3.058	0.230
**Chronic diseases**			
No	References		
Yes	1.667	0.476–5.837	0.424
**PSQI items**			
**Subjective sleep quality**	1.260	0.962–1.652	0.093
**Sleep latency**	1.170	0.936–1.462	0.169
**Sleep duration**	1.121	0.919–1.367	0.261
**Habitual sleep efficiency**	1.126	0.901–1.408	0.297
**Sleep disturbance**	2.112	1.408–3.169	0.0001***
**Use of sleep medications**	1.526	1.160–2.008	0.003**
**Daytime dysfunction**	1.766	1.381–2.257	0.0001***
**Global PSQI score**	1.133	1.062–1.208	0.0001***
**Global PSQI score category**			
Normal sleep quality (< 5)	Reference		
Poor sleep quality (≥ 5)	2.837	1.408–5.716	0.0001***

## Discussion

This study demonstrates that video gaming remains a problem among young people in Saudi Arabia, including medical students. Abnormal gamers were identified in 38.8% of the study population, and abnormal gaming was linked to poor sleep quality when comparing abnormal gamers with normal gamers (92% vs. 80.3%). It also revealed that out of all types of abnormal gamers, addicted gamers often relied on sleep medication to help them sleep at night and took longer time to fall asleep (*p* = 0.050 and *p* = 0.045, respectively).

This is one of the few studies to report the frequency of video gamers among Saudi medical students [14, 15]. A study by Al Asqah et al. has used the special IGD 9-Item Short Scale consisting of 9 yes-or-no questions; the scale assigns one point for each “yes” answer, and a score of 2 to 4 indicates a risky gamer and 5 or more a disordered gamer. The authors revealed that the prevalence of IGD was 8%, and 19.3% were risky gamers [14]. Another study, however, reported that the prevalence of IGD was 4.6%, using a different questionnaire [15]. Moreover, Khrad et al. found a prevalence of 10.1% of gaming addiction among students in various Saudi Arabian universities [16]. Furthermore, worldwide the prevalence of gaming addiction concurs with these local data. Shrestha et al. reported a prevalence of 8.5% of gaming disorder among medical students in Nepal [17]. Ohayon et al. also reported a prevalence of 5.3% among university students in the United State of America [18]. This study assessed addiction using the DSM-5 recommendation. Rehbein et al., however, reported a prevalence of only 1.16% of IGD according to the DSM-5 recommendation among German children aged 13–18 years [19]. All of these studies reported a prevalence lower than that found in our population. There are several factors actually that may contribute to this discrepancy. This could be explained by the difference in the scales used to assess gaming addiction. GAS requires lower scores to classify gamers as abnormal compared to the IGD-20 and the DSM-5 criteria. In addition, the differences in the studied populations, as adults typically have easier access to gaming devices and the financial means to purchase video games compared to children, who are often dependent on their parents for such resources. Furthermore, another explanation involves the effect of the Coronavirus disease of 2019 (COVID-19) pandemic, which has significantly increased sales in the gaming industry. Additionally, as people spend more time playing various types of video games, they discover new games that they can enjoy both online and offline.

Recent studies have confirmed that gamers increased their playing time during the pandemic, due to the lockdown and stress that accompanied the pandemic, but they have not tracked changes in the time spent playing games following the pandemic [2, 12, 16, 17]. Therefore, further research is needed to explore the severity of gaming addiction among individuals who developed it during the pandemic and to determine whether any changes have occurred in their playing habits after the pandemic. Our study assessed gaming habits over a 6-months period, between January and June 2023, which is almost 2 years after the COVID-19 pandemic. Hence, it is hard to appreciate the effect of the pandemic on our findings although it is expected that it has enhanced the addiction as a result of having more time and verities to play during the pandemic.

After applying the regression analysis, the GAS dataset (Table 4) revealed that among gamers with poor sleep quality, the factors with greatest impact on abnormal gaming were mood modification (playing games to forget about real life), relapse (unsuccessful attempts to reduce the time spent on games by themselves or others), and problems (neglecting important activities to play games). As expected, mood modification and problems adversely affected abnormal gaming (Exp(B) values of 1.565 and 2.041, respectively), whereas relapse had a protective effect (Exp(B) value of 0.487). The latter probably reflects the value of frequent counselling as its effect takes time to work.

Although there was no difference between the type of abnormal gamers regarding the frequency of poor sleep quality, however, addicted gamers were more frequent users of sleep medication as they have longer sleep latency. However, after applying the regression model to the PSQI dataset (Table 5), we found that sleep disturbance, the use of sleep medications, and daytime dysfunction were significant risk factors for poor sleep quality among abnormal gamers. Compared with similar studies that explored PSQI components, these risk factors have also been observed in other populations [12, 20]. A Pakistani study, showed that in addition to sleep disturbance and daytime dysfunction, sleep duration and sleep efficiency are risk factors for poor sleep quality among gamers as determined by the PSQI [12]. Furthermore, a study conducted in Taiwan, reported that significant risk factors for poor sleep quality among internet users were subjective sleep quality, sleep latency, sleep disturbance, and daytime dysfunction [20]. Moreover, the use of sleep medication as a risk factor distinguished our population from the Pakistani and Taiwanese populations. The use of sleep medications would improve sleep duration and reduce sleep latency in an attempt to improve sleep efficiency and hence the quality of sleep. This may explain the differences in risk factors to poor sleep quality in our study compared to others. Finally, Demographic characteristics, including age, sex, housing type, and housing tenure were not significant risk factors for poor sleep quality or abnormal video gaming.

Of note, GAS is probably a wider test for abnormal gaming and hence, it was preferred to IGD-20 in our study. Several studies have used the 20-item IGD test (IGD-20) instead of GAS [4, 5, 8]. While both tests feature the same items, the IGD-20 focuses solely on online games, whereas the GAS is broader in scope, because gaming addiction can occur in both online and offline games. Online games are usually difficult to pause, and medical students have busy schedules during their academic year, making it difficult for them to be frequent users of online games. In addition, online games require a stable internet connection, which can be difficult to achieve in university campuses. In contrast, offline games are available on multiple handheld devices, such as smartphones, tablets, and laptops, and can be played anywhere and anytime without being restricted to the Internet.

While video gaming can offer several benefits for medical students, such as enhanced cognitive skills and stress relief, it is important to manage gaming habits carefully to avoid potential negative impacts on physical and mental health. This can be achieved by instructing all mentors to periodically explain to students the cons and pros of video gaming. Also, celebrating awareness days regarding healthy life style that includes normal use of gaming would help in avoiding all consequences of abnormal gaming. Such consequences include poor time management, interfering with study time, clinical duties, as well as negatively affecting sleep quality. Furthermore, prolonged gaming sessions can contribute to a sedentary lifestyle, increasing the risk of weight gain and obesity. Additionally, extended screen time can cause eye strain, headaches, and fatigue, affecting overall health and productivity. More seriously excessive gaming can lead to gaming addiction, which has been associated with increased levels of anxiety and depression [21]. Recently, Rajab et al. assessed the relationship between gaming addiction and stress in 2675 adolescents and found that those who were addicted were more likely to experience stress than those who were not addicted (11.4% of the total population had high stress and 5% were addicted to video games), and those who were addicted were less likely to score excellent grades (38.5% vs. 51.2%) [4].

There are few limitations in our study. The data were collected using a self-reported online survey and its accuracy depends on the subjective response of the participants. Since GAS evaluates the items over a 6-month period, the accuracy of the results might be affected by the recall bias. Additionally, medical students receive more than five surveys daily on their social media accounts and experience survey fatigue, as a result, the response rate is expected to be low. Finally, this study did not assess the participants’ GPAs to determine whether addiction was related to or affected student performance.

## Data Availability

No datasets were generated or analysed during the current study.

## Abbreviations

DSM-5: The Diagnostic and Statistical Manual of Mental Disorders,
GAS: Gaming Addiction Scale
IGD: Internet gaming disorder
PSQI: Pittsburgh Sleep Quality Index
